# Detecting Microsatellites in Genome Data: Variance in Definitions and Bioinformatic Approaches Cause Systematic Bias

**DOI:** 10.4137/ebo.s420

**Published:** 2008-02-09

**Authors:** Angelika Merkel, Neil J. Gemmell

**Affiliations:** School of Biological Sciences, University of Canterbury, Christchurch, New Zealand

**Keywords:** microsatellites, short tandem repeats, definition, genome, array length, study bias

## Abstract

Microsatellites are currently one of the most commonly used genetic markers. The application of bioinformatic tools has become common practice in the study of these short tandem repeats (STR). However, *in silico* studies can suffer from study bias. Using a meta-analysis on microsatellite distribution in yeast we show that estimates of numbers of repeats reported by different studies can differ in the order of several magnitudes, even within a single genome. These differences arise because varying definitions of microsatellites, spanning repeat size, array length and array composition, are used in different search paradigms, with minimum array length being the main influencing factor. Structural differences in the implemented search algorithm additionally contribute to variation in the number of repeats detected. We suggest that for future studies a consistent approach to STR searches is adopted in order to improve the power of intra- and interspecific comparisons

## Introduction

Microsatellites or short sequence/tandem repeats (SSRs/STRs) are tandemly repeated DNA sequences of (commonly) 1–6bp length per repeat unit. Their high length polymorphism and abundance in all genomes make them the genetic marker of choice for a diverse range of applications spanning linkage analysis and genetic mapping through to forensics and ecological and evolutionary studies (Goldstein and Schlötterer, 1999). Interest in microsatellite mutational dynamics is increasing, with significant interest emerging in the use of genomic data to investigate the evolution of these ubiquitous and useful sequences. To date, a significant number of studies have investigated microsatellite abundance in a range of species in order to examine the evolution of these simple sequences and infer their functional roles, if any, in gene regulation, genome structure etc. (Kashi and King, 2006). Putative distribution biases have been investigated for introns, exons and intergenic regions as well as possible associations with other genomic elements, such as interspersed repeats (Arcot et al. 1995; Li et al. 2004; Lim et al. 2004; Malpertuy et al. 2003; Toth et al. 2000).

However, comparisons among large scale *in silico* genome studies, even from the same genomic data, are fraught with methodological bias. A recent paper by Leclercq et al. (2007) outlines significant differences among search algorithms based on intrinsic structure of the search algorithm and the parameter settings. We present a meta-analysis on microsatellite distribution in yeast as an example on how divergent study results can be in practice. We confirm Leclercq’s (2007) findings, but more importantly we show that the differences are rooted in a long-lived controversy, ever since microsatellites were first discovered 20 years ago; how exactly to define a microsatellite. Interspecies comparisons that derive from different studies are particularly vulnerable to erroneous conclusions, and it is an intricate task to tease out the patterns of microsatellite evolution from those arising from study bias.

## Methods

We undertook a meta-analysis of the published literature on microsatellite distribution in the yeast genome (*Saccharomyces cerevisiae*). The studies chosen are all comparisons of microsatellite distribution patterns (motif, size class, and array length) that include *S. cerevisiae* as one of the focal species, but differ in the approach and software used to detect microsatellite sequences (Table 1).

## Results

**Figure 1:**
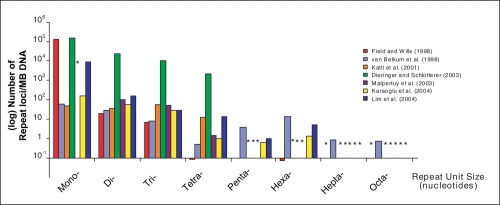
Microsatellite distribution in *S. cerevisiae*. Histogram shows the number of repeat loci per size class reported by each study. For details on parameter settings see Supplementary Table 1). *no data available.

All analyzed studies confirm unique species-specific motif distribution patterns and an over-representation of long arrays over short arrays, which is in concordance with current models of microsatellite evolution. However, we find striking differences in the reported results (Figure 1). For example, Dieringer and Schlotterer, (2003) report more repeats across all motif types than others, up to several magnitudes difference. This study scored repeat frequencies (loci/Mbp) in the order of 104 for di- and trinucleotides and 103 for tetranucleotides, compared to 102 for dinucleotides and 101 for tri-and tetranucleotides, which are the next highest frequencies out of all other studies. Among all repeat sizes, mononucleotides are especially variable in the numbers of loci reported. We found frequency counts that ranged from a minimum of 46 loci/Mbp (Katti, Ranjekar, and Gupta, 2001) to a maximum of 142,200 loci/Mbp (Dieringer and Schlotterer, 2003). The relative abundance of size classes also differs among studies. For example, all studies report mononucleotides as the most abundant size class with decreasing frequencies of longer repeat units, except Katti et al. (2001) who report the highest numbers for trinucleotides and van Belkum et al. (1998) who show an increased frequency for penta- and hexanucleotides.

## Discussion

Given that the seven studies we examined have essentially analyzed the same genome data (small variations in build version not withstanding) for the same range of motifs, it is surprising to see such wide divergence in results. Here we discuss, that the crux of the problem derives from the different definitions of microsatellites used in each study. Differences in characteristics such as array length, unit size and purity inevitably transcribe into deviations in the parameter settings used in bioinformatic search tools, which subsequently lead to large discrepancies in results.

### Minimum array length

Historically, the preferred size for microsatellites selected as genetic markers has been a minimum of five repeats (Selkoe and Toonen, 2006). However, the minimum array length required for strand slippage to occur is much lower. Rose and Falush, (1998) determined a critical length at around eight nucleotides based on microsatellite distribution in yeast, while Lai and Sun, (2003) approximated a minimum threshold of four copies for di-, tri-, tetra-, penta- and hexanucleotides and at least nine copies for mononucleotides for humans. In practice, however, the actual *in silico* detection of short repeats may be restricted by the minimum resolution of the search algorithm, e.g. 10 or 11 nucleotides in the case of Tandem Repeats Finder (Benson, 1999) used by Malpertuy et al. (2003). Within our meta-analysis the differences in minimum cut-off length explain most of the variance: studies applying a low length threshold, e.g. in the case of mononucleotides around 2–5bp (Dieringer and Schlotterer, 2003; Field and Wills, 1998; Lim, et al. 2004), harvest high repeat frequencies, whereas studies applying a higher threshold of 10 or 20bp report far fewer microsatellites (Karaoglu et al. 2005; Katti et al. 2001; van Belkum et al. 1998) (see Table 1).

### Repeat unit size

Di-, tri- and tetranucleotide repeats dominate the literature because they have been found most frequently in the genome and are useful genetic markers (Jarne and Lagoda, 1996). Mononucleotides, whilst common, have been largely avoided as they cause problems during amplification (Selkoe and Toonen, 2006). However, from a mechanistic point of view, microsatellites are characterized by high levels of length polymorphism caused by DNA strand slippage, which can occur in repeat arrays composed of units that range from 1 to ~10bp in length (Armour et al. 1999; Jeffreys et al. 1994; Levinson and Gutman 1987b; Sia et al. 1997). Definitions of the motif length required to constitute a microsatellite vary in the literature: i.e. 1–6bp (Goldstein and Pollock, 1997), 1–5bp (Chambers and MacAvoy, 2000), 2–6bp (Schlotterer et al. 1998), or even 2–8bp (Armour et al. 1999). The same spread is reflected in our study survey: out of seven analyzed studies, one study excludes mononucleotide repeats (Malpertuy, Dujon, and Richard, 2003), only four studies report numbers for penta- and hexanucleotides, and only one examines hepta- and octa-nucleotides (van Belkum et al. 1998) (see Table 1 for search parameters).

### Purity and internal structure of the array

So far, the majority of *in silico* searches have investigated only perfect microsatellites as they are computationally easier to detect. However, perfect microsatellites are not the only type of microsatellites. In fact, a repeat array might be classified as perfect (identical copies), imperfect (mismatches and indels are allowed) or compound/complex (array includes different motifs) (Buschiazzo and Gemmell, 2006; Chambers and MacAvoy, 2000). For most of the recent repeat detection tools, the level of imperfection can be varied as a parameter within the search. Despite this, Katti et al. (2001) and Malpertuy et al. (2003) are the only studies in our survey that allowed imperfections: a mismatch every 10th nucleotide, and succeeding mismatches after the first five perfect copies, respectively. While the available data do not allow us to detect a correlation between more or less stringent search criteria and high or low reported microsatellite frequencies, it appears logical that the inclusion or exclusion of imperfections in search parameters will influence the results of genomic comparisons.

### Computational approach and genome build

There are additional, more subtle variables in the search that are rooted within the bioinformatic approach itself. Peculiarities of the underlying algorithm, such as combinatorial treatment of repeats in the identification procedure and/or redundancy filtering of overlaps or internal repetitions, may profoundly affect the overall pattern reported. Within our dataset, four studies (Katti et al. 2001; Lim et al. 2004; Malpertuy et al. 2003; van Belkum et al. 1998) apply the same minimum length threshold of 20bp in the case of tetranucleotides, but report frequencies of 0.5, 1.5, 12.6 and 13 repeats/Mbp, respectively. Comparing the documentation for the search approaches (Table 1) suggests that studies using different algorithmic approaches report varying repeat frequencies. Unfortunately, details of parameter settings and the structure of the applied algorithm are not consistently published, thereby precluding detailed comparisons.

Different sequence builds and the inclusion of the mitochondrial genome (mtDNA) in the sequence analyzed can also contribute to variation in results. We ran TRF in default mode on three different *S. cerevisiae* genome builds and found no significant variation in the total numbers, types and distributions of the microsatellites reported (Supplement 1). However, a significantly higher frequency of microsatellites was detected within the mitochondrial genome compared to the nuclear genome (Supplement 2) and the inclusion or exclusion of this genome in comparisons would result in a modest difference between studies.

## Conclusion

The issue of how to exactly define a microsatellite is a long argued subject, upon which researchers have not yet reached consensus. Differences in parameters used in repeat detection, especially minimum array length, lead to large systematic biases in study results, where variations in microsatellite frequency can reach the extent of several magnitudes among studies even within the same genome.

Several authors have put forward microsatellite definitions, varying mainly based on their research background. First, describing types of repeats with respect to the degradation and complexity of the array subdivisions can be quite specific, such as in forensic and medicine (Urquhart et al. 1994), focusing on mutational behaviors of individual loci and alleles. We are predominately concerned with genomic analysis and propose therefore only three types of microsatellite spanning mono-hexanucleotides: perfect (repeat copies 100% identical), imperfect (mismatches and indels incorporated) and complex/compound (consist of several motifs, potentially with mismatches). Second, minimum array length has been traditionally defined by the occurrence of strand slippage events and the extent of the resulting microsatellite polymorphism. This has led to analyses employing either stacked thresholds that depend on repeat size (for example see Table 1) or length classes, e.g. microsatellites class I: 12 < 20nt, microsatellite class II: >20nt (Temnykh et al. 2001). We suggest the following thresholds to start with, after Lai and Sun (2003): 12nt for mono-trinucleotides, 16nt for tetranucleotides, 20nt for pentanucleotids and 24nt for hexanucleotides. Absolute minimum thresholds for slippage events, tend to be group specific (between 8–15nt) and need to be adjusted individually for each species to eliminate background noise, i.e. random occurrences of microsatellites, from true over- or under representation.

Ideally, future studies ensure that all data are gathered and analyzed in a consistent manner, which should enable a consensus approach to emerge within the literature. However, due to the potential intricacies of microsatellite distribution in different genomic architectures, this might not always be possible in an absolute manner. Therefore, we encourage all authors to report their parameter settings and algorithms in detail (including the underlying reasoning), to enable sensible comparisons across studies. The importance of the issue can not be emphasized enough in the genomic era, where cross-species comparisons are the tools of trade.

## Supplementary Material

**Table S1:** Variation in TRF results* between genome builts

**Date genome built**	1/01/1998	1/10/2003	30/11/2006
**Total sequence size (nuclear), nt**	12069303	12070521	12070899
**Repeats found with TRF (default)**	406	407	406

* TRF default parameters: 2 7 7 80 10 50 6 (minimum length: 25nt)

Figure S1 Varition in microsatellite abundance between different chromosome and mtDNA (↓). Note the roughly linear relationship between loci number and chromosome size with mtDNA (↓) as outlier.
Sequences were downloaded from ftp at SGD (ftp://genome-ftp.stanford.edu/pub/yeast/sequence/NCBI_genome_source).

## Figures and Tables

**Table 1:** Studies utilized in the meta-analysis. All studies report comparisons of microsatellite distribution pattern in yeast. Table shows (from left to right) study, algorithm or software employed, the type of repeat that was investigated (with respect to perfection/imperfection) and parameter that were implemented in the bioinformatics search, such as repeat size (mono-octanucleotide) and array length (minimum/maximum threshold).

Study	Algorithm	Type of repeat	Repeat parameters
Field and Wills (1998)	PERL script–regular expression^1^	Perfect repeats	All mononucleotides: 1–42bp Repeat size: 2, 3, 4, 5, 6bp Minimum length: 16, 24, 32, 40, 48, 56, 64bp
van Belkum et al. (1998)	C-script^2^	Perfect repeats	Repeat size: 1, 2, 3, 4, 5, 6, 7, 8bp Minimum length: 10, 10, 18, 20, 18, 20, 21, 24bp
Katti Ranjekar and Gupta (2001)	C-script, –base-by-base search using adjacent sliding windows for alignments	Imperfect repeats (mismatch every 10th nt)	Repeat size: 1, 2, 3, 4bp Minimum length: 20, 20, 21, 20bp
Dieringer and Schlötterer (2003)	C-script,–motif search for consecutive sequence stretches	Perfect repeats (incl. partial copies)	Repeat size: 1, 2, 3, 4bp Minimum length: 2, 4, 6, 8bp Maximum length: 20bp
Malpertuy, Dujon and Richard (2003)	TRF software (Benson 1999), –statistic/heuristic approach	Imperfect repeats (match: (+1) mismatch: (−2, −3, 4) indels: (−6, −9, −12))	Pattern size: 2, 3, 4bp Minimum length: 10, 15, 20bp Maximum length: 20 repeats
Karaoglu, Lee and Meyer (2005)	PYTHON script	Perfect repeats	Pattern size: 1, 2, 3, 4, 5, 6bp Minimum length: 10bp
Lim et al. (2004)	C++ script,–base-by-base search using adjacent sliding windows for alignment	Perfect repeats	Pattern size: 1, 2, 3, 4, 5, 6bp Minimum length: 5 repeats

1 Personal communication, algorithm is now implemented as *MsatFinder* software (http://www.bioinf.ceh.ac.uk/msatfinder/).

2 The URL address given for the server was not valid anymore at the time of our study, no further information could be found.

## Abbreviations

### Abbreviations

nt: nucleotide
kb: kilo base
