# Supplementary figures and images for: Detecting Microsatellites in Genome Data: Variance in Definitions and Bioinformatic Approaches Cause Systematic Bias
Source: Evol Bioinform Online. 2008 Feb 9;4:1–6. doi: 10.4137/ebo.s420 (PMC2614199; doi:10.4137/ebo.s420)

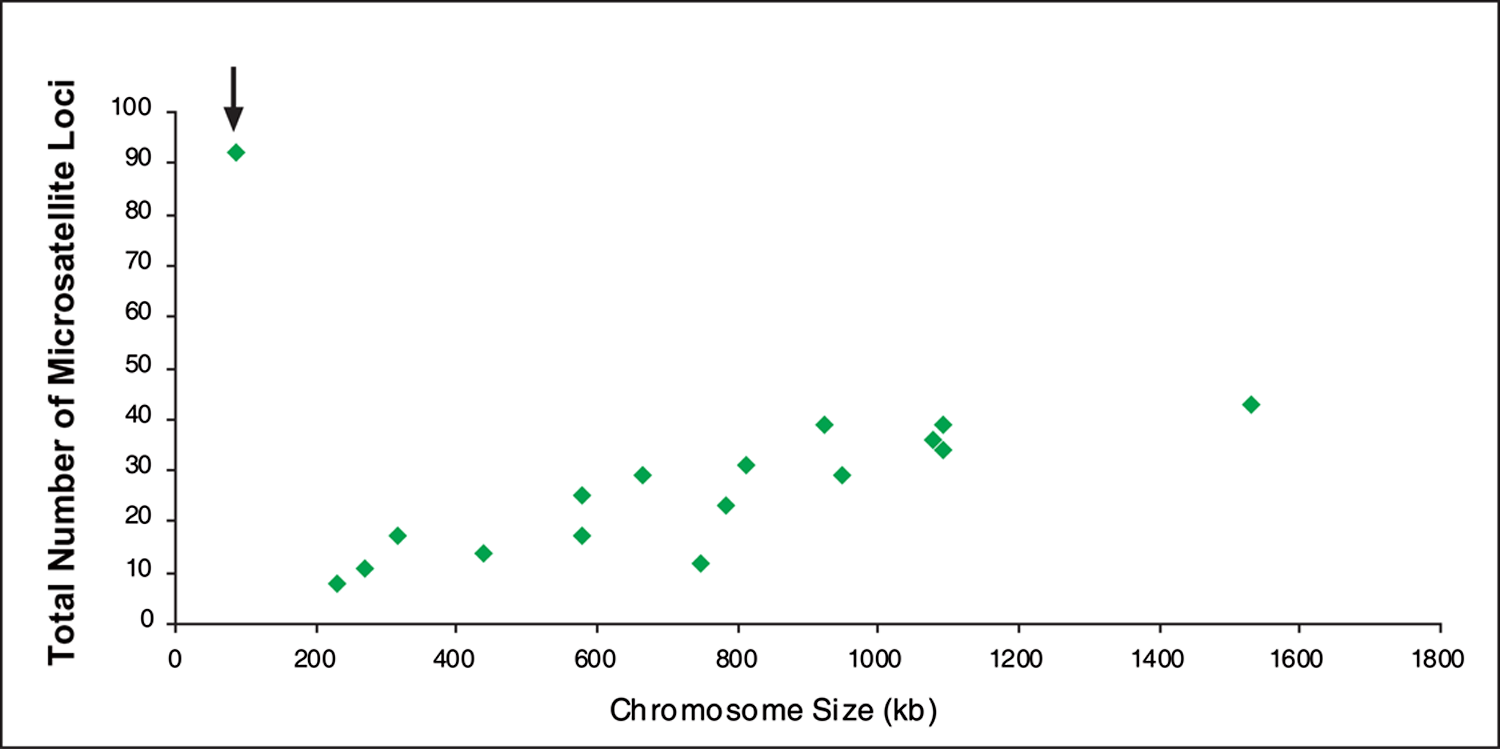

Supplement: Figure S1 — Varition in microsatellite abundance between different chromosome and mtDNA (↓). Note the roughly linear relationship between loci number and chromosome size with mtDNA (↓) as outlier. Sequences were downloaded from ftp at SGD (ftp://genome-ftp.stanford.edu/pub/yeast/sequence/NCBI_genome_source). [file ebo-04-001-s02.tif]
